# Supplementary material for: High-sensitive nascent transcript sequencing reveals BRD4-specific control of widespread enhancer and target gene transcription
Source: Nat Commun. 2023 Aug 17;14:4971. doi: 10.1038/s41467-023-40633-y (PMC10435483; doi:10.1038/s41467-023-40633-y)

Supplementary Fig. 1

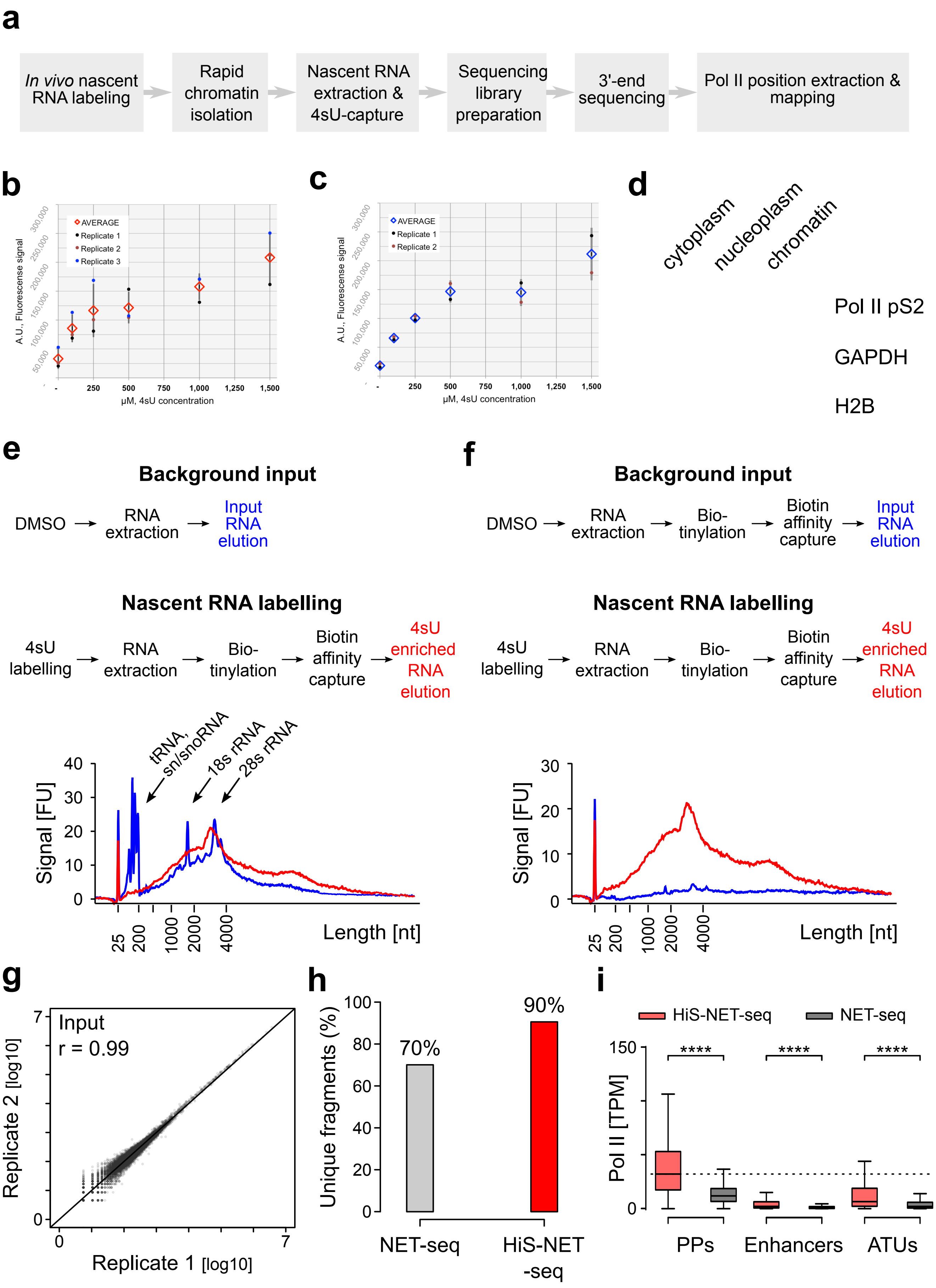

Supplementary Fig. 1

j

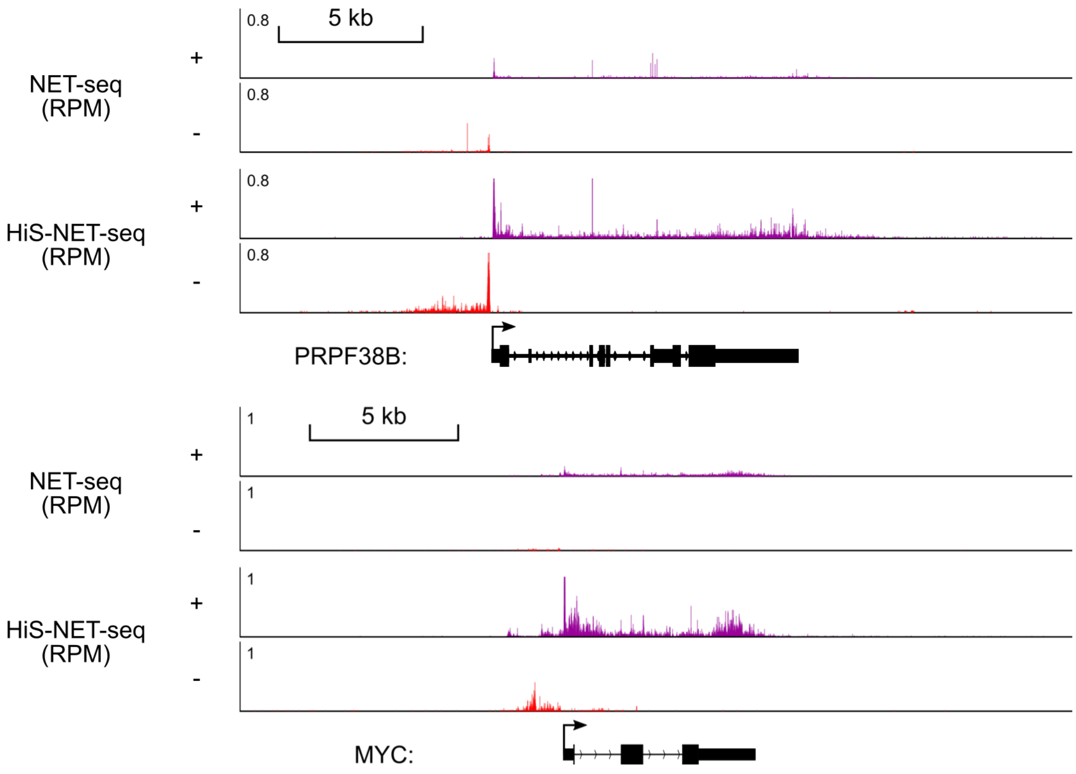

k

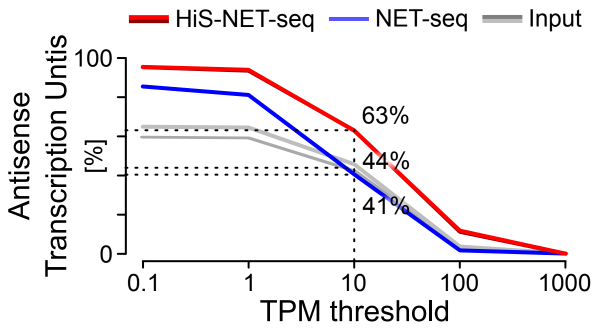

Supplementary Fig. 1: Quality control measurements for the HiS-NET-seq approach.

(a) Overview of the main steps of the HiS-NET-seq approach. The high sensitivity of the method is achieved by combining the quantitative isolation of chromatin-associated RNA with a brief 4sU pulse and the affinity enrichment of 4sU-labeled RNA.

(b-c) Optimization of 4sU incorporation in K562 and NIH/3T3 cells. Human K562 **(b)** and murine NIH/3T3 **(c)** cells were exposed for 10 minutes to different concentrations of 4sU, followed by the extraction of chromatin-bound RNA, biotinylation of the incorporated 4sU and dot blotting for biotin. A concentration of 500 µM of 4sU was selected for all following experiments as an additional increase in the concentration did not lead to a higher labeling efficiency. Source data are provided as a Source Data file.

(d) Rapid cell fractionation efficiently enriches actively transcribing Pol II. Immunoblot for Serine 2-phosphorylated Pol II as well as marker proteins of the cytoplasmic (GAPDH) and the chromatin (histone 2B, H2B) fraction. >90% of p-Ser2 Pol II was captured in the chromatin fraction. Experiment was performed in duplicate. Source data are attached.

(e-f) HiS-NET-seq captures nascent Pol II transcripts with high selectivity. Schematic view of the main steps of the nascent RNA labeling procedure and the procedure for obtaining non-labeled nascent RNA (background, DMSO control). To monitor the selectivity of the purification of nascent Pol II transcripts, nascent RNA was extracted from cells grown in the presence of 4sU or DMSO (control). **(e)** Analysis of the RNA length distribution using a Bioanalyzer (Agilent) revealed the relative depletion of Pol I and Pol III-transcribed short RNA (rRNA, tRNA, snRNA, snoRNA), highlighted by the arrow, from the 4sU-enriched nascent RNA. **(f)** Non-4sU-labeled RNA was not recovered by affinity capture as presented by the RNA length distributions.

(g) High reproducibility of input samples from unlabelled chromatin fractions. Pearson's correlation analysis of Pol II occupancy per active gene for two biological input measurements (r = 0.99, 2h DMSO) in K562 cells. The input lacks the 4sU labeling step of the HiS-NET-seq protocol. Human gene counts were RLE-normalized, and 0.5 pseudo counts were added.

(h) HiS-NET-seq libraries reveal a higher complexity compared to standard NET-seq. Fraction of unique fragments obtained with standard NET-seq and HIS-NET-seq (average from two independent biological replicate measurements) for K562 cells.

(i) Boxplot quantification of TPM normalized Pol II occupancy at indicated regions (two-sided Wilcoxon rank sum test; ****: $p<2.2e-16$) using HiS-NET-seq and NET-seq, respectively. Element types as described in the Methods include promoter-proximal regions (PPs) of active genes (n = 9,454), FANTOM5 enhancers (n=6,313), and antisense transcription units (ATUs, n=22,367). See Fig. 3d legend for boxplot definition.

(j) Two gene examples showing RPM normalized Pol II occupancy measured by standard NET-seq (one replicate) and HiS-NET-seq (two merged biological replicates) in K562 cells.

(k) Quantification of Pol II occupancy measured at antisense transcription units (n = 22,367) by HiS-NET-seq and NET-seq, respectively. Depicted are the percent of transcribed elements with indicated TPM threshold or higher.

Supplementary Fig. 2

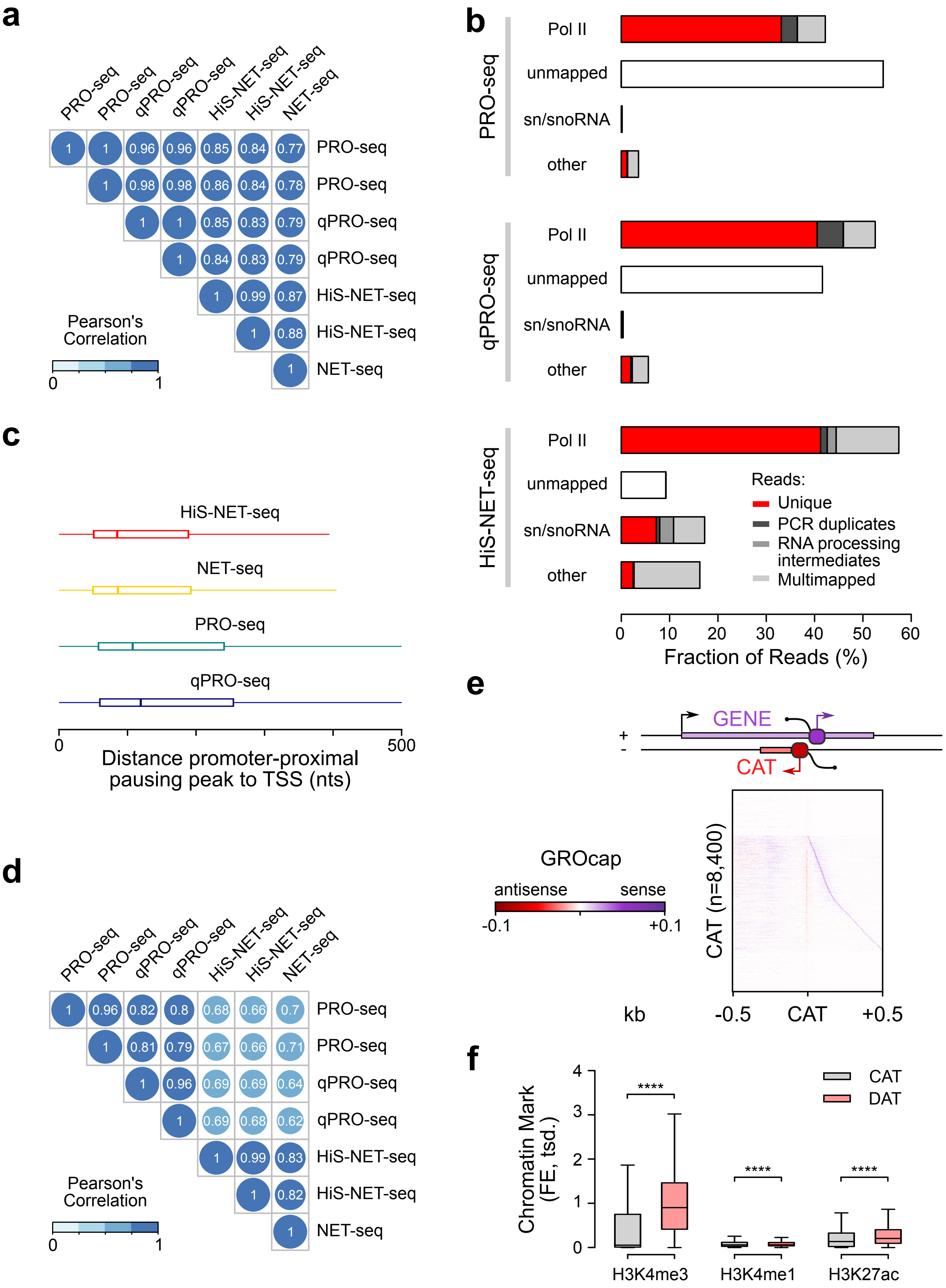

Supplementary Fig. 2: Comparative analyses of HiS-NET-seq, NET-seq, PRO-seq and qPRO-seq data.

(a, d) Correlation analyses of PRO-seq, qPRO-seq, standard NET-seq and HiS-NET-seq data at **(a)** active genes and **(d)** promoter-proximal regions (TSS to TSS + 0.3 kb). Pairwise comparisons of Pearson's correlation between one standard NET-seq replicate and two biological replicates of PRO-seq, qPRO-seq [1] and HiS-NET-seq. Pol II occupancy data was obtained for K562 cells. Human counts were RLE-normalized, and 0.5 pseudo counts were added. Excluded were signal outliers above the 99.9-quantile.

(b) Barplot shows the fraction of sequencing reads that mapped to Pol II transcribed regions (described in methods), no genomic region, sn/snoRNA genes, and other regions. Other regions include miRNAs, as well as transcribed regions of Pol I, Pol III, and mitochondrial RNA polymerase. Fractions are indicated for PRO-seq (top panel), qPRO-seq (middle panel), and HiS-NET-seq (lower panel) data. Data sets were randomly sampled as described in *'Comparison of HiS-NET-seq, PRO-seq, and qPRO-seq data'* of the methods section.

(c) Boxplot quantifications show the mean distance of the pausing peak to the TSS in nucleotides (nts) across biological replicates for one standard NET-seq replicate and two biological replicates of PRO-seq, qPRO-seq [1] and HiS-NET-seq. The maxima position in the promoter-proximal region (TSS to TSS + 0.5 kb) was considered for each active gene (n=10,483). See Fig. 3d legend for boxplot definition.

(e) RPM normalized GRO-cap data [2] at CAT units with a TPM <5. The center marks the 5'-end of CAT regions as identified by HiS-NET-seq.

(f) Boxplot quantification shows the mean fold enrichment (FE) for two biological replicates of the indicated chromatin marks in the upstream TSS region (TSS - 0.3 kb to TSS) of CAT

(n=14,881) and DAT (n=5,970) sites (two-sided Wilcoxon rank sum test ****: $p < 5e\text{-}16$). See Fig. 3d legend for boxplot definition.

Supplementary Fig. 3

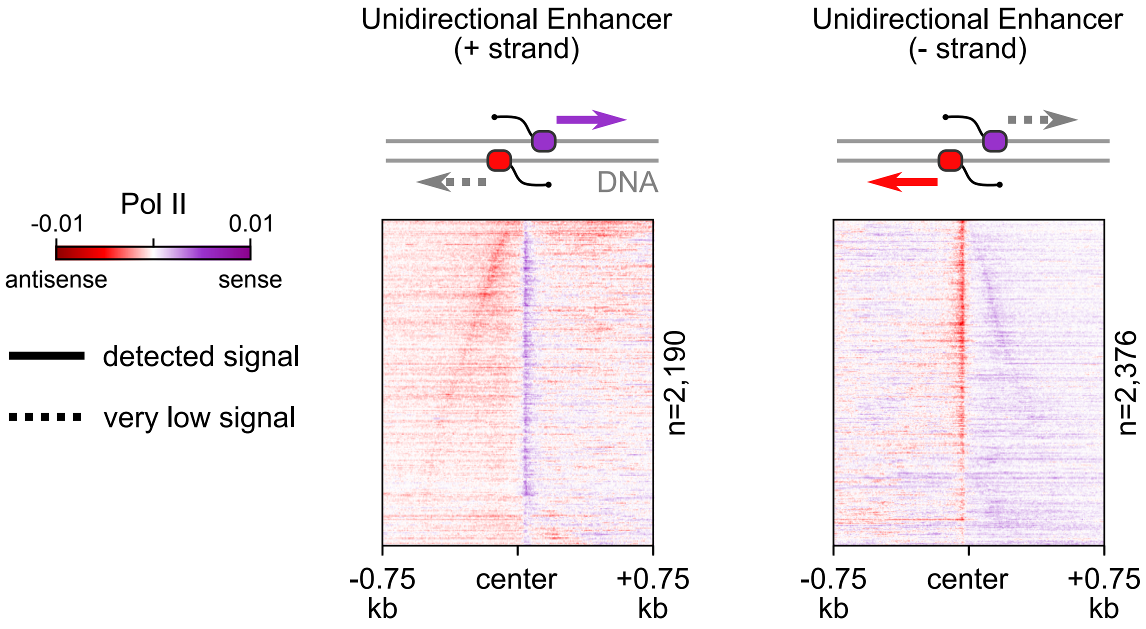

Supplementary Fig. 3: Bi-directional enhancer transcription escapes automated detection at lowly transcribed regions.

Heat map representation of RPM normalized Pol II occupancy measured by HIS-NET-seq for individual nucleotides at a subset of putative enhancer regions. Transcription regions were detected for one direction (solid arrow line) at the (+) (left) or (-) (right) strand, respectively. The transcription units at the opposite strands (dashed arrow line) dropped below the detection threshold and remained undetected for the enhancer detection algorithm. Although most of the bi-directional transcription signals could be observed, these enhancers were classified by the algorithm as uni-directionally transcribed enhancers.

Supplementary Fig. 4

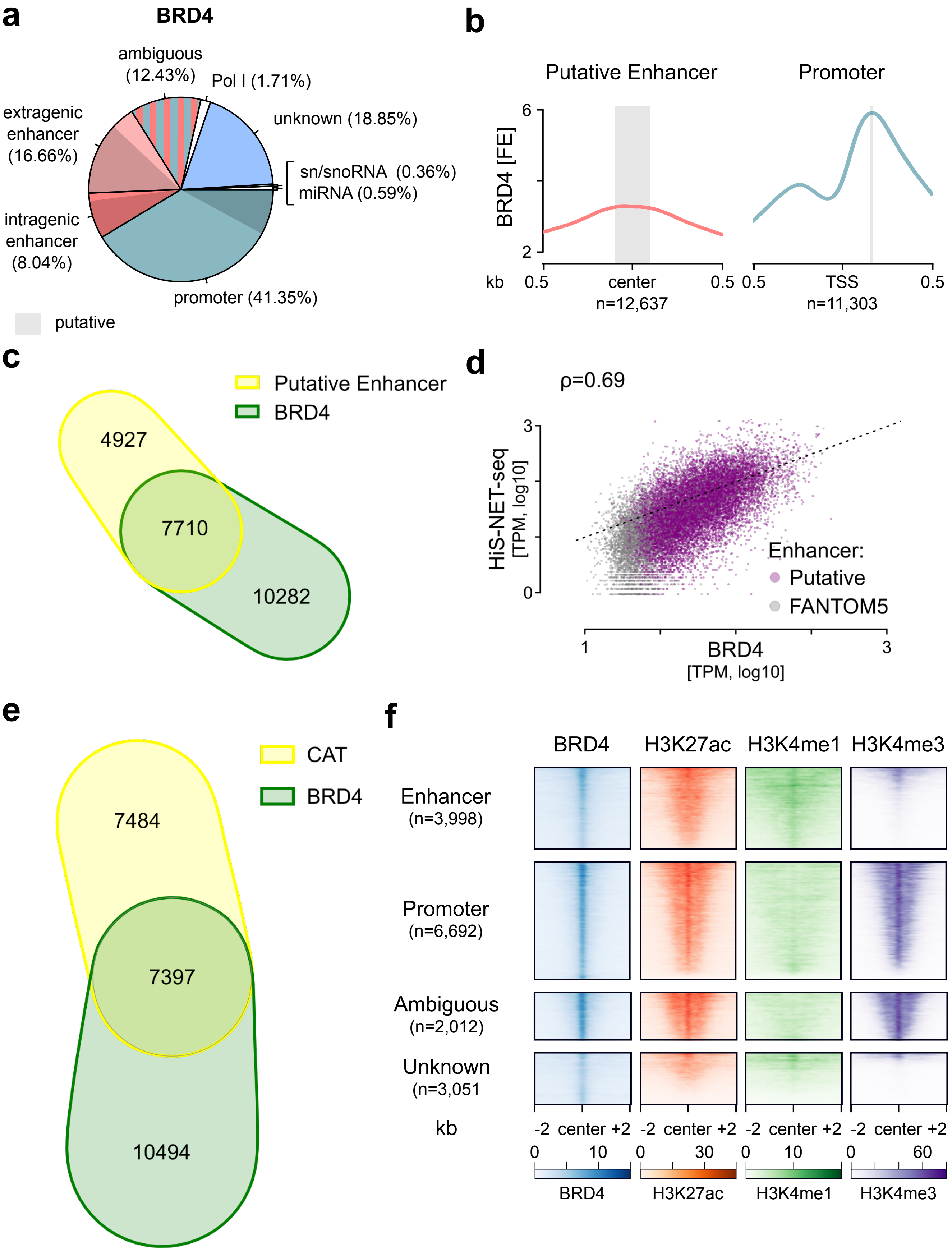

Supplementary Fig. 4: Genome-wide BRD4 binding correlates with transcription at putative enhancer regions.

(a) BRD4 binds enhancer and promoter regions. The pie chart shows the classification of robust BRD4 peaks identified by two biological replicates in K562 cells. The classification considers the GENCODE promoter and K562 FANTOM5 enhancer regions. Furthermore, we added putative enhancer regions (gray) identified by the HiS-NET-seq enhancer detection approach. BRD4 peaks that cover both enhancer and promoter regions are classified as ambiguous.

(b) BRD4-occupancy profile for enhancer and promoter regions. Meta-gene profiles of BRD4 occupancy (fold enrichment (FE) over matched input control) as obtained for K562 cells by ChIP-Rx measurements (DMSO 2h). Presented are 5'-regions of active genes (right) and central regions of putative enhancers (left) as identified by HiS-NET-seq. Average profiles are depicted from two biological replicate measurements. A gray box marks the peak occupancy locations of BRD4 at enhancer (+/- 100 nt) and promoter (+ 160 nt) regions, respectively. TSS: transcription start site.

(c) Putative enhancer regions are bound by BRD4. Venn diagram depicts the overlap between putative enhancer regions identified by HiS-NET-seq and robustly (two biological replicates) detected BRD4 binding sites by ChIP-Rx in K562 cells.

(d) Enhancer transcription and BRD4 occupancy strongly correlate. The scatterplot shows the correlation between Pol II (HiS-NET-seq) and BRD4 occupancy at enhancer regions (Spearman's rank correlation coefficient: ρ=0.69). Depicted are mean TPM values (log10) across two biological replicate measurements at FANTOM5 (n=6,313, gray) and putative HiS-NET-seq (n=12,025, purple) enhancer regions.

(e) BRD4 binds convergent antisense transcription (CAT) units. Venn diagram depicts the overlap between CAT units identified by HiS-NET-seq and robustly (two biological replicates) detected BRD4 binding sites by ChIP-Rx in K562 cells.

(f) Enhancer-associated BRD4 binding sites lack H3K4me3. Heatmaps visualize FE-normalized signals of BRD4 occupancy and histone marks [3] in K562 dTAG-BRD4 and K562 cells. Depicted are BRD4 binding sites identified by ChIP-Rx that are associated with enhancer, promoter, ambiguous (promoter and enhancer regions), and unknown loci.

Supplementary Fig. 5

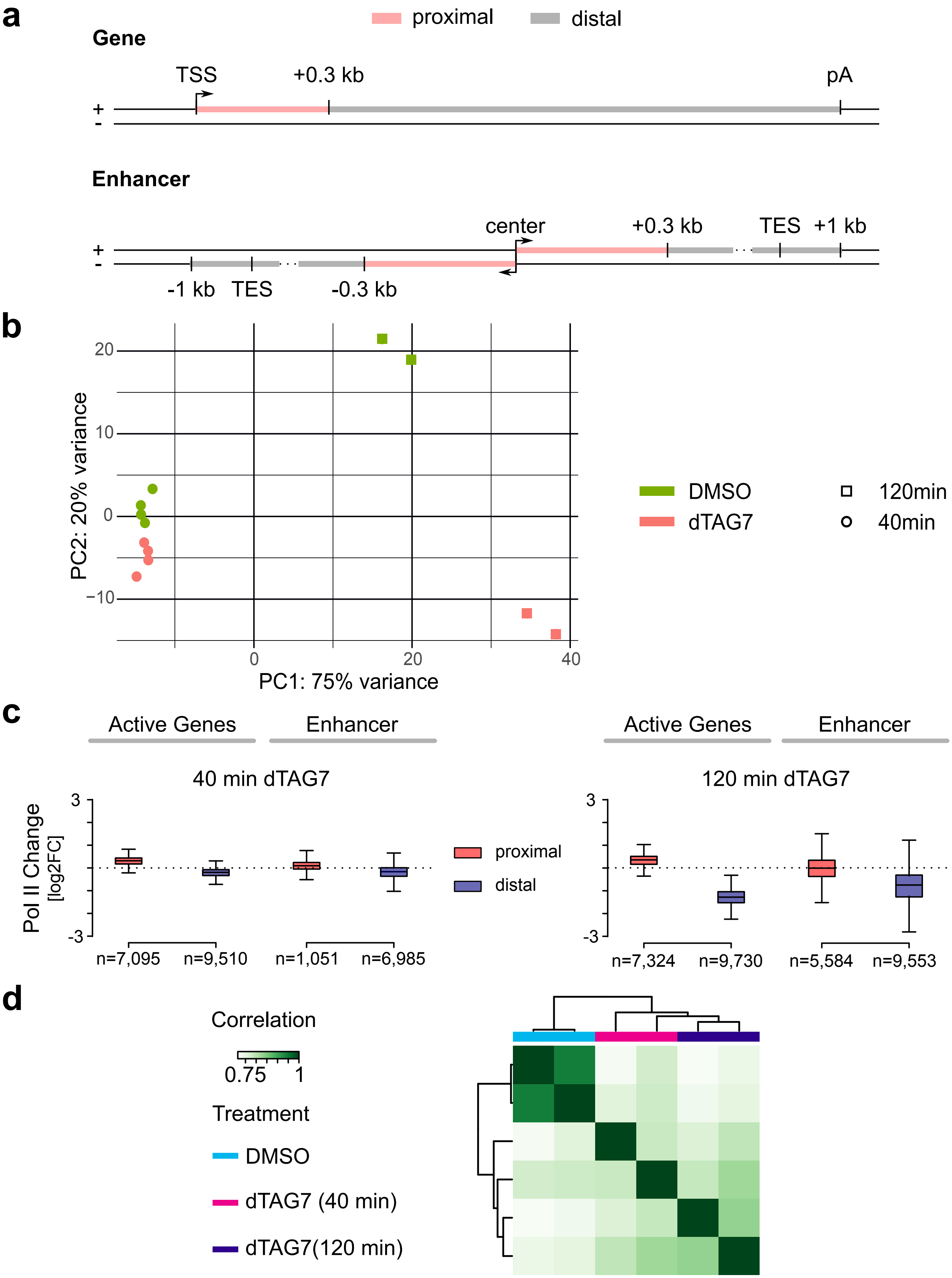

Supplementary Fig. 5: BRD4 controls elongation activation at genes and enhancers.

(a) The scheme depicts proximal (pink) and distal (gray) gene and enhancer regions. For genes, we refer to those regions as promoter-proximal and gene-body regions. The definition is relative to the transcription start site (TSS) and the polyadenylation site (pA). For enhancers, the definition is relative to the center point and, if possible, the respective transcription end sites (TESs) at both strands. Uni-directional enhancer regions lack transcription at the opposite strand. -1 kb and +1 kb refer to the respective TES.

(b) Principal component analysis of HiS-NET-seq data shows clustering of perturbation experiments. The principal component analysis reduces the complex HIS-NET-seq samples from a high dimension into a two-dimensional space. Depicted are Pol II gene occupancy measurements at active human genes in K562 from indicated treatment types (control (DMSO); BRD4-specific degradation (dTAG7)) and treatment times (40 and 120 minutes). Data is corrected using spike-in normalization. Furthermore, we corrected systematic bias introduced by different batches using the 'removeBatchEffect' function from limma [4]. This approach removes any shifts in the data that can be explained by the batch.

(c) Acute BRD4 ablation has similar effects on enhancer and gene transcription. Boxplot quantification of Pol II occupancy changes (log2) measured by HiS-NET-seq upon 40 (left panel) and 120 (right panel) minutes of dTAG7 treatment at distal and proximal regions of active genes and putative enhancers. See Fig. 3d legend for boxplot definition.

(d) Hierarchical clustering of BRD4 occupancy data. Pearsons's correlation for the spike-in normalized BRD4 ChIP-Rx samples which were obtained for K562 dTAG-BRD4 cells using DiffBind [5]. The data samples are separated by condition using hierarchical clustering.

Supplementary Fig. 6

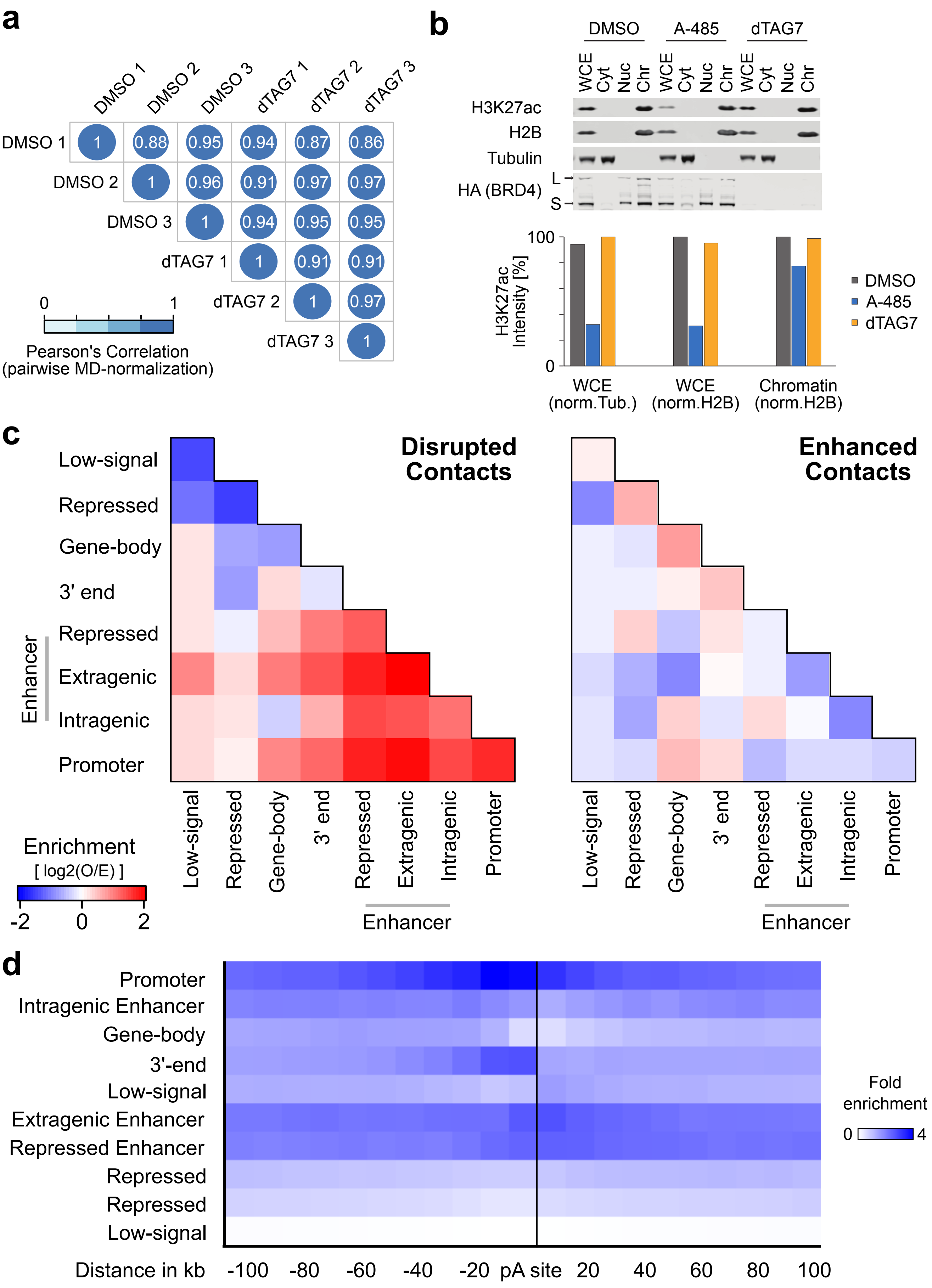

Supplementary Fig. 6: Acute BRD4 ablation reduces interaction frequency between regulatory regions.

(a) HiChIP replicates strongly correlate. Pearson correlation analysis is shown for pairwise comparisons between 3D genome interaction frequencies obtained from H3K27ac HiChIP measurements in K562 cells. Data was pairwise normalized using the MD-normalization strategy implemented in the HiCcompare package [6].

(b) Western blot analysis using whole cell extract (WCE) and subcellular fractions of K562 dTAG-BRD4 cells. Cells were treated with DMSO (control), 10 µM A-485 (HAT inhibitor) or 500 nM dTAG7 for 2 hours. Signal intensity of H3K27ac was normalized to both Tubulin (Tub) and histone 2B (H2B). Experiment was performed in duplicate. Source data are provided as a Source Data file and in the attachment. WCE, whole cell extract; Cyt, cytoplasm, Nuc, nucleoplam; Chr, chromatin, HA, hemagglutinin epitope tag. S refers to the short isoform and L to the long isoform of BRD4.

(c) 3D contacts among regulatory interactions are over proportionally disrupted. The heat map shows over- and under-represented interaction types among disrupted and increased 3D interactions after 2h of BRD4 degradation in K562. Different Interaction types exist depending on the genomic regions that are contacting each other. Enrichment was calculated as the logarithmic ratio of observed (O) and expected (E) contacts. We used the genome-wide distribution of all pairwise 3D interactions to calculate the expected values.

(d) Enrichment of chromHMM states at polyA sites. The heat map presents the fold enrichment of indicated chromHMM [7] states in K562 cells relative to the polyA sites of annotated RefSeq genes.

Method	Description	Detection of Pol II occupancy	Source
Gro-CAP / Pro-CAP	Gro-CAP and Pro-CAP are variants of GRO-seq and PRO-seq which rely on cap selection and sequencing from the 5'-end of the labeled RNA to capture the genomic position of active TSSs at nucleotide-resolution. Compared to HiS-NET-seq, Gro-CAP and Pro-CAP cannot map RNA polymerase density at genomic target sites.		[2], [8]
mNET-seq	As compared to HiS-NET-seq mNET-seq relies on Micrococcal digest of isolated chromatin and antibody-based enrichment of solubilized RNA polymerase transcription complexes along with the nascent RNA. The 3'-ends of the purified nascent RNA are converted into a sequencing library by an mNET-seq-specific library preparation procedure. Compared to the mNET-seq protocol, the HiS-NET-seq library preparation uses unique molecular identifiers (UMIs), spike-in controls and a control step that reduces the risk of reverse transcription artifacts.	✓	[9]
Nano-COP / nascONT-seq	Nano-COP and nascONT-seq involve Oxford Nanopore sequencing of long nascent RNA to monitor the timing and patterns of co-transcriptional *RNA* processing. In contrast to HiS-NET-seq, nano-cop and nascONT-seq include an enzymatic tailing step to prepare the 3'-ends on nascent RNAs for Oxford nanopore sequencing. Consequently, newly tailed nascent RNAs and naturally polyadenylated chromatin-bound mature RNAs are sequenced through the nanopore. In case of nano-COP tailed RNAs are directly sequenced whereas in nascONT-seq tailed RNAs are converted into cDNA prior to nanopore long-read sequencing. Compared to HiS-NET-seq, Nano-COP and nascONT-seq exhibit lower sensitivity due to lower throughput of Nanopore sequencing.	✓	[10], [11], [12]
NET-CAGE	Cap analysis of gene expression (CAGE) methods enable the detection and usage of TSSs with single-nucleotide and DNA strand resolution genome-wide. Compared		[13]

	to HiS-NET-seq, NET-CAGE relies on the purification of 5'-capped chromatin-bound RNAs and cDNA sequencing of the original 5'-ends of the capped RNAs. Similar to Pro-CAP/Gro-CAP, CAGE methods can not reveal the genomic density of engaged RNA polymerase along genomic target regions.		
PRO-seq / qPRO-seq	PRO-seq and qPRO-seq are nuclear run-on based RNA polymerase profiling methods which make use of labeling of nascent RNA with biotin-NTPs to measure the genomic position of transcribing RNA polymerase with nucleotide resolution. Compared to HiS-NET-seq, PRO-seq/qPRO-seq require to hold transcription and re-initiate transcription in the presence of biotin-NTPs after nuclei have been prepared. Nuclear run-on methods may fail to capture a subset of engaged RNA polymerases that can't resume transcription under the labeling conditions. As compared to HiS-NET-seq, classic PRO-seq protocols also lack spike-in controls and UMIs.	✓	[1], [8], [14]
SLAM-seq	SLAM-seq relies on the chemical conversion of labeled RNA to measure time-resolved RNA synthesis and degradation kinetics. Compared to HiS-NET-seq, SLAM-seq does not capture 3'-nascent RNA ends of engaged RNA polymerase and therefore cannot reveal the density of transcribing Pol II.		[15]
TT-seq / TT_{chem}-seq	TT-seq/TT_{chem}-seq involves the isolation of labeled RNA to map transcriptionally active regions and monitor RNA synthesis. Compared to HiS-NET-seq, TT-seq/TT_{chem}-seq does not exclusively capture 3'-nascent RNA ends of engaged RNA polymerase and therefore cannot reveal the density of transcribing Pol II.		[16], [17]

Supplementary Table 1: Comparison of HiS-NET-seq with other genomic transcription analysis methods that have been used to identify actively transcribed genes and enhancers.

Approaches that also reveal the Pol II occupancy at target genomic locations are indicated (3rd column). Except of mNET-seq and NET-CAGE all methods are based on metabolic labeling of nascent chromatin-associated or newly synthesized RNA.

Supplemental References

1. Judd, J., Wojenski, L.A., Wainman, L.M., Tippens, N.D., Rice, E.J., Dziubek, A., Villafano, G.J., Wissink, E.M., Versluis, P., Bagepalli, L., et al. (2020). A rapid, sensitive, scalable method for Precision Run-On sequencing (PRO-seq). Biorxiv, 2020.05.18.102277. 10.1101/2020.05.18.102277.

2. Core, L.J., Martins, A.L., Danko, C.G., Waters, C.T., Siepel, A., and Lis, J.T. (2014). Analysis of nascent RNA identifies a unified architecture of initiation regions at mammalian promoters and enhancers. Nat Genet *46*, 1311–1320. 10.1038/ng.3142.

3. Dunham, I., Kundaje, A., Aldred, S.F., Collins, P.J., Davis, C.A., Doyle, F., Epstein, C.B., Frietze, S., Harrow, J., Kaul, R., et al. (2012). An integrated encyclopedia of DNA elements in the human genome. Nature *489*, 57–74. 10.1038/nature11247.

4. Ritchie, M.E., Phipson, B., Wu, D., Hu, Y., Law, C.W., Shi, W., and Smyth, G.K. (2015). limma powers differential expression analyses for RNA-sequencing and microarray studies. Nucleic Acids Res *43*, e47–e47. 10.1093/nar/gkv007.

5. Stark, R., and Brown, G. (2011). DiffBind: differential binding analysis of ChIP-Seq peak data. Bioconductor *R package version 100.4.3*. 10.18129/b9.bioc.diffbind.

6. Stansfield, J.C., Cresswell, K.G., Vladimirov, V.I., and Dozmorov, M.G. (2018). HiCcompare: an R-package for joint normalization and comparison of HI-C datasets. Bmc Bioinformatics *19*,

279. 10.1186/s12859-018-2288-x.

7. Ernst, J., and Kellis, M. (2012). ChromHMM: automating chromatin-state discovery and characterization. Nat Methods 9, 215–*216*. 10.1038/nmeth.1906.

8. Mahat, D.B., Kwak, H., Booth, G.T., Jonkers, I.H., Danko, C.G., Patel, R.K., Waters, C.T., Munson, K., Core, L.J., and Lis, J.T. (2016). Base-pair-resolution genome-wide mapping of active RNA polymerases using precision nuclear run-on (PRO-seq). Nat Protoc 11, 1455–1476. 10.1038/nprot.2016.086.

9. Nojima, T., Gomes, T., Grosso, A.R.F., Kimura, H., Dye, M.J., Dhir, S., Carmo-Fonseca, M., and Proudfoot, N.J. (2015). Mammalian NET-Seq Reveals Genome-wide Nascent Transcription Coupled to RNA Processing. Cell *161*, 526–540. 10.1016/j.cell.2015.03.027.

10. Drexler, H.L., Choquet, K., and Churchman, L.S. (2020). Splicing Kinetics and Coordination Revealed by Direct Nascent RNA Sequencing through Nanopores. Mol Cell 77, 985-998.e8. 10.1016/j.molcel.2019.11.017.

11. Drexler, H.L., Choquet, K., Merens, H.E., Tang, P.S., Simpson, J.T., and Churchman, L.S. (2021). Revealing nascent RNA processing dynamics with nano-COP. Nat Protoc 16, 1343–1375. 10.1038/s41596-020-00469-y.

12. Arnold, M., Bressin, A., Jasnovidova, O., Meierhofer, D., and Mayer, A. (2021). A BRD4-mediated elongation control point primes transcribing RNA polymerase II for 3′-processing and termination. Mol Cell 81, 3589-*360*3.e13. 10.1016/j.molcel.2021.06.026.

13. Hirabayashi, S., Bhagat, S., Matsuki, Y., Takegami, Y., Uehata, T., Kanemaru, A., Itoh, M., Shirakawa, K., Takaori-Kondo, A., Takeuchi, O., et al. (2019). NET-CAGE characterizes the dynamics and topology of human transcribed cis-regulatory elements. Nat Genet *51*, 1369–1379. 10.1038/s41588-019-0485-9.

14. Kwak, H., Fuda, N.J., Core, L.J., and Lis, J.T. (2013). Precise Maps of RNA Polymerase Reveal How Promoters Direct Initiation and Pausing. Science *339*, 950–953. 10.1126/science.1229386.

15. Muhar, M., Ebert, A., Neumann, T., Umkehrer, C., Jude, J., Wieshofer, C., Rescheneder, P., Lipp, J.J., Herzog, V.A., Reichholf, B., et al. (2018). SLAM-seq defines direct gene-regulatory functions of the BRD4-MYC axis. Science 360, 800–805. 10.1126/science.aao2793.
16. Schwalb, B., Michel, M., Zacher, B., Frühauf, K., Demel, C., Tresch, A., Gagneur, J., and Cramer, P. (2016). TT-seq maps the human transient transcriptome. Science *352*, 1225–1228. 10.1126/science.aad9841.
17. Gregersen, L.H., Mitter, R., and Svejstrup, J.Q. (2020). Using TTchem-seq for profiling nascent transcription and measuring transcript elongation. Nat Protoc 15, 604–627. 10.1038/s41596-019-0262-3.
18. Ney, P.A., Sorrentino, B.P., McDonagh, K.T., and Nienhuis, A.W. (1990). Tandem AP-1-binding sites within the human beta-globin dominant control region function as an inducible enhancer in erythroid cells. Gene Dev 4, 993–1006. 10.1101/gad.4.6.993.

Source Data Supplementary Fig. 1d

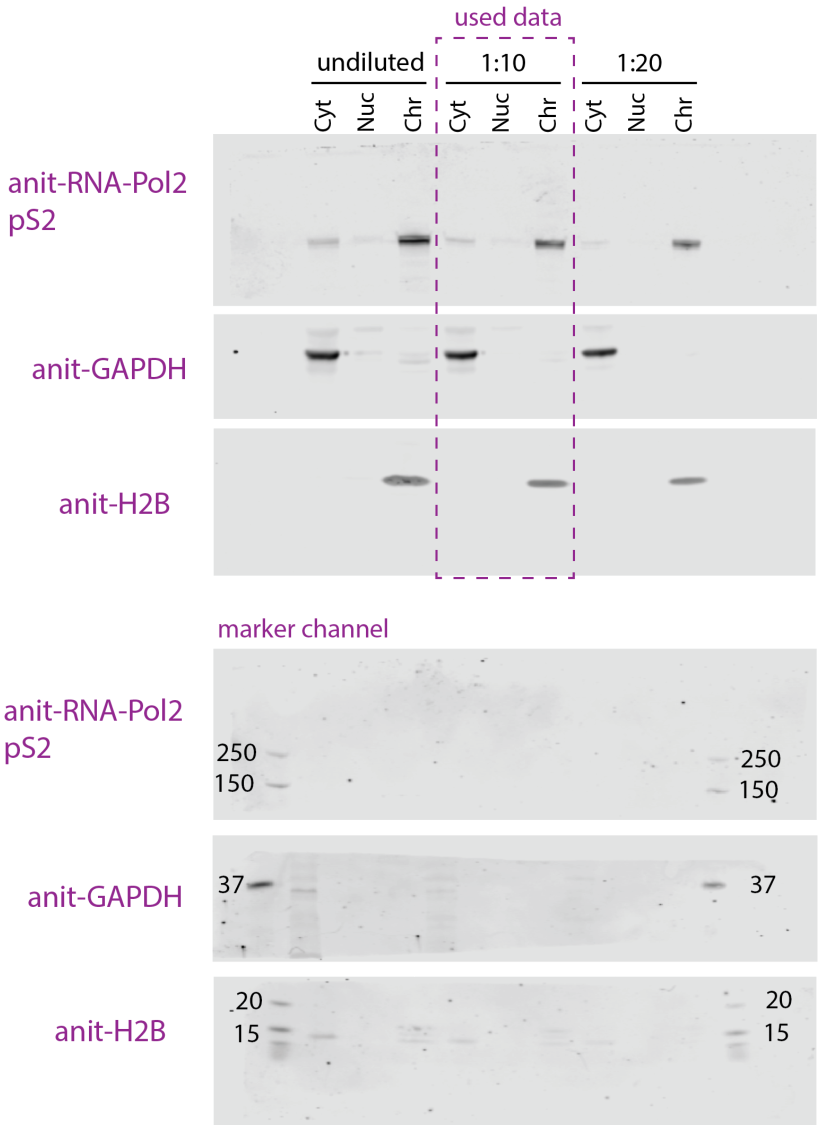

Source Data Supplementary Fig. 6b

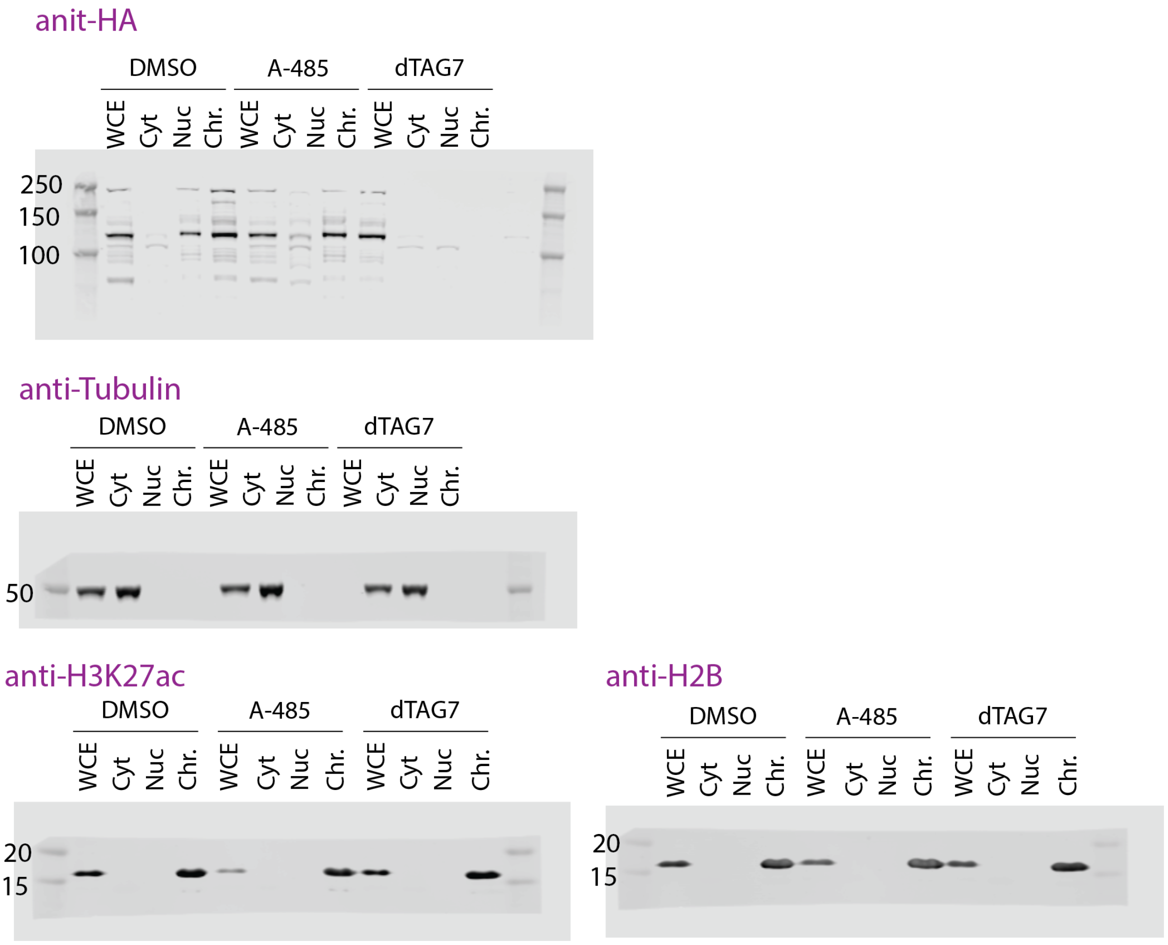

Supplement: Supplementary file 1 — Supplementary Information [file 41467_2023_40633_MOESM1_ESM.pdf]
