# Supplementary material for: High-sensitive nascent transcript sequencing reveals BRD4-specific control of widespread enhancer and target gene transcription
Source: Nat Commun. 2023 Aug 17;14:4971. doi: 10.1038/s41467-023-40633-y (PMC10435483; doi:10.1038/s41467-023-40633-y)
Supplement: Supplementary file 3 — Description of Additional Supplementary Files [file 41467_2023_40633_MOESM3_ESM.pdf]

Description of Additional Supplementary Files

File Name: Supplementary Data 1
Description: Putative extragenic enhancer regions. The file contains putative extragenic enhancer regions identified after pooling all control HiS-NET-seq replicates (GSM6612461-GSM6612462, GSM6612467-GSM6612468, GSM6612473-GSM6612476) in human K562 and K562 dTAG-BRD4 cells. Selected were extragenic transcription units with H3K27ac and H3K4me1 histone marks. The table format follows the BED12 (https://bedtools.readthedocs.io/en/latest/content/general-usage.html) standard. The file contains uni-directional and bi-directional extragenic enhancers. For uni-directional enhancers, the extragenic transcript with strand information is given. In contrast, bi-directional extragenic enhancers span two divergently oriented transcription units. The projected genomic positions of enhancer centers are listed in the 5th column.

File Name: Supplementary Data 2
Description: List of convergent and divergent antisense transcription units identified by HiS-NET-seq. The file contains convergent antisense transcription (CAT) and divergent antisense transcription (DAT) units identified after pooling all control HiS-NET-seq replicates (GSM6612461-GSM6612462, GSM6612467-GSM6612468, GSM6612473-GSM6612476) in human K562 and K562 dTAG-BRD4 cells. CAT and DAT units are defined relative to the corresponding active gene (ID, 5th column), as shown in Fig. 2b. The table format follows the BED (https://bedtools.readthedocs.io/en/latest/content/general-usage.html) standard.

File Name: Supplementary Data 3
Description: Putative intragenic enhancer regions. The file contains putative intragenic enhancer regions identified after pooling all control HiS-NET-seq replicates (GSM6612461-GSM6612462, GSM6612467-GSM6612468, GSM6612473-GSM6612476) in human K562 and K562 dTAG-BRD4 cells. CAT units listed in Supplementary Data 1 and that were not associated with promoters but had H3K27ac and H3K4me1 histone marks were selected (see STAR Methods). The table format follows the BED12 (https://bedtools.readthedocs.io/en/latest/content/general-usage.html) standard.

File Name: Supplementary Data 4
Description: Genomic DNA sequences of putative enhancers tested in the enhancer reporter assay. The file contains the tested constructs, their genomic location and size (bp: base pairs). The positive control enhancer 'HS2 minimal' sequence was obtained from Ney et al. [18].

File Name: Supplementary Data 5
Description: Significant BRD4 interactors. Proteins were significantly enriched by native BRD4 immunoprecipitation and identified by mass spectrometry for the K562 dTAG-BRD4 cell line. Isotype-matched normal IgG was used to estimate the specificity of the enrichment. Only protein groups identified by $\geq$ 2 peptides in $\geq$ 70% of the replicates were considered. Significance was determined by a two-tailed Student's T-test. Imputed values are not shown.

File Name: Supplementary Data 6
Description: Enhancer cloning primer sequences. This file contains forward and reverse primers used for the generation of both sense (S) and antisense (AS) enhancer constructs that were employed for Gibson assembly with the pGL3 promoter vector. In addition, forward and reverse primers are listed for the amplification of the enhancer constructs used for conventional restriction enzyme cloning.
