# Supplementary material for: High-sensitive nascent transcript sequencing reveals BRD4-specific control of widespread enhancer and target gene transcription
Source: Nat Commun. 2023 Aug 17;14:4971. doi: 10.1038/s41467-023-40633-y (PMC10435483; doi:10.1038/s41467-023-40633-y)
Supplement: Supplementary file 10 — Reporting Summary [file 41467_2023_40633_MOESM10_ESM.pdf]

nature portfolio

Corresponding author(s): Dr. Andreas Mayer

Last updated by author(s): Jul 7, 2023

# Reporting Summary

Nature Portfolio wishes to improve the reproducibility of the work that we publish. This form provides structure for consistency and transparency in reporting. For further information on Nature Portfolio policies, see our Editorial Policies and the Editorial Policy Checklist.

## Statistics

For all statistical analyses, confirm that the following items are present in the figure legend, table legend, main text, or Methods section.

| n/a | Confirmed | |
|---|---|---|
| ☐ | ☒ | The exact sample size ($n$) for each experimental group/condition, given as a discrete number and unit of measurement |
| ☐ | ☒ | A statement on whether measurements were taken from distinct samples or whether the same sample was measured repeatedly |
| ☐ | ☒ | The statistical test(s) used AND whether they are one- or two-sided<br>*Only common tests should be described solely by name; describe more complex techniques in the Methods section.* |
| ☐ | ☒ | A description of all covariates tested |
| ☐ | ☒ | A description of any assumptions or corrections, such as tests of normality and adjustment for multiple comparisons |
| ☐ | ☒ | A full description of the statistical parameters including central tendency (e.g. means) or other basic estimates (e.g. regression coefficient) AND variation (e.g. standard deviation) or associated estimates of uncertainty (e.g. confidence intervals) |
| ☒ | ☐ | For null hypothesis testing, the test statistic (e.g. $F$, $t$, $r$) with confidence intervals, effect sizes, degrees of freedom and $P$ value noted<br>*Give P values as exact values whenever suitable.* |
| ☒ | ☐ | For Bayesian analysis, information on the choice of priors and Markov chain Monte Carlo settings |
| ☒ | ☐ | For hierarchical and complex designs, identification of the appropriate level for tests and full reporting of outcomes |
| ☐ | ☒ | Estimates of effect sizes (e.g. Cohen's $d$, Pearson's $r$), indicating how they were calculated |

*Our web collection on statistics for biologists contains articles on many of the points above.*

## Software and code

Policy information about availability of computer code

Data collection: We used the software fasterq-dump v3.0.0 to collect data.

Data analysis: Data processing and analysis uses the following software.
1. R v.4.2.2 packages:
- BiocParallel
- DESeq2 v1.38.3
- GenomicRanges v1.50.2
- HiCcompare v1.20.0
- PerformanceAnalytics v2.0.4
- RColorBrewer v1.1-3
- VennDiagram v1.7.3
- corrplot v0.92
- data.table v1.14.8
- ggplot2 v3.4.2
- patchwork v1.1.2
- pheatmap
- plyr
- viridis v0.6.2

2. R v.4.0.5 packages:

- DiffBind v3.0.15

3. R v3.6.3 packages:
- nVennR v0.2.3

4. Python v3.10.8 packages:
- pyBigWig v0.3.18
- numpy 1.24.3
- Bio v1.81
- matplotlib.pylab v3.6.3
- pandas 2.0.2
- pickle5 NA
- pyBigWig v0.3.22
- pybedtools 0.9.0
- pysam 0.21.0

5. Stand-alone software:
- gunzip v1.12,
- bedGraphToBigWig v.4,
- bigWigToBedGraph NA,
- bedtools v2.30.0,
- deeptools v3.2.1,
- samtools v1.16.1,
- STAR v2.7.3a,
- FastQC v0.11.9,
- cutadapt v4.1,
- starcode v1.1
- chromHMM v1.19

6. We further provide share custom computer code at https://github.molgen.mpg.de/MayerGroup/HiS-NET-seq_paper_code.git and upon request.

For manuscripts utilizing custom algorithms or software that are central to the research but not yet described in published literature, software must be made available to editors and reviewers. We strongly encourage code deposition in a community repository (e.g. GitHub). See the Nature Portfolio guidelines for submitting code & software for further information.

## Data

Policy information about availability of data

All manuscripts must include a data availability statement. This statement should provide the following information, where applicable:

- Accession codes, unique identifiers, or web links for publicly available datasets
- A description of any restrictions on data availability
- For clinical datasets or third party data, please ensure that the statement adheres to our policy

HiS-NET-seq, standard NET-seq, HiChIP and ChIP-Rx data generated in this study have been deposited at the Gene Expression Omnibus (GEO) database under super series accession number GSE214594 [https://www.ncbi.nlm.nih.gov/geo/query/acc.cgi?acc=GSE214594]. The mass spectrometry proteomics data generated in this study have been deposited in the ProteomeXchange Consortium via the PRIDE partner repository under accession code PXD043652. Publicly available datasets with the following accession numbers were used in this study: GSE158963 [https://www.ncbi.nlm.nih.gov/geo/query/acc.cgi?acc=GSE158963] (SI-NET-seq K562 dTAG-BRD4, DMSO 2h, dTAG7 2h), GSE150625 [https://www.ncbi.nlm.nih.gov/geo/query/acc.cgi?acc=GSE150625] (qPRO-seq K562 and PRO-seq K562), GSE60456 [https://www.ncbi.nlm.nih.gov/geo/query/acc.cgi?acc=GSE60456] (GRO-cap K562), GSE158965 [https://www.ncbi.nlm.nih.gov/geo/query/acc.cgi?acc=GSE158965] (ChIP-Rx K562 dTAG-BRD4 PAF1, DMSO 2h, dTAG7 2h), ENCSR109IQO [https://www.encodeproject.org/experiments/ENCSR109IQO/] (Total RNA-seq K562), ENCSR000CLW [https://www.encodeproject.org/experiments/ENCSR000CLW/] (RNA-seq NIH/3T3), ENCSR000AKP [https://www.encodeproject.org/experiments/ENCSR000AKP/] (ChIP-seq K562 H3K27ac), ENCSR000EWC [https://www.encodeproject.org/experiments/ENCSR000EWC/] (ChIP-seq K562 H3K4me1), ENCSR000EWA [https://www.encodeproject.org/experiments/ENCSR000EWA/] (ChIP-seq K562 H3K4me3), ENCSR000EWB [https://www.encodeproject.org/experiments/ENCSR000EWB/] (ChIP-seq K562 H3K27me3), ENCSR000AKR [https://www.encodeproject.org/experiments/ENCSR000AKR/] (ChIP-seq K562 H3K36me3), ENCSR000APD [https://www.encodeproject.org/experiments/ENCSR000APD/#!] (ChIP-seq K562 H3K79me2). We used the human and mouse genome reference (GRCh38.p12 [https://ftp.ebi.ac.uk/pub/databases/gencode/Gencode_human/release_28/GRCh38.primary_assembly.genome.fa.gz]; GRCm38.p6 [https://ftp.ebi.ac.uk/pub/databases/gencode/Gencode_mouse/release_M18/GRCm38.primary_assembly.genome.fa.gz]) and annotation (v28 [https://ftp.ebi.ac.uk/pub/databases/gencode/Gencode_human/release_28/gencode.v28.primary_assembly.annotation.gtf.gz]; M18 [https://ftp.ebi.ac.uk/pub/databases/gencode/Gencode_mouse/release_M18/gencode.vM18.primary_assembly.annotation.gtf.gz]) provided by gencode. K562 enhancer were extracted from HACER [http://bioinfo.vanderbilt.edu/AE/HACER/download/T1.txt]. Source data are provided with this paper.

## Human research participants

Policy information about studies involving human research participants and Sex and Gender in Research.

| | |
|---|---|
| Reporting on sex and gender | Sex and gender are irrelevant for this study investigating general transcriptional regulatory events. The female cell lines K562 and K562 dTAG-BRD4 were used. |
| Population characteristics | Does not apply |
| Recruitment | Does not apply |

| | |
|---|---|
| Ethics oversight | Does not apply |

Note that full information on the approval of the study protocol must also be provided in the manuscript.

# Field-specific reporting

Please select the one below that is the best fit for your research. If you are not sure, read the appropriate sections before making your selection.

☒ Life sciences ☐ Behavioural & social sciences ☐ Ecological, evolutionary & environmental sciences

For a reference copy of the document with all sections, see nature.com/documents/nr-reporting-summary-flat.pdf

# Life sciences study design

All studies must disclose on these points even when the disclosure is negative.

| | |
|---|---|
| Sample size | We used no statistical method to estimate the sample size. For sequencing-based approaches, we choose the number of biological replicates according to the recommendations from the original publications for HiChIP (Mumbach et al., 2016) and ChIP-Rx (Orlando et al., 2014). Subsequently, for each condition, we produced three biological replicates for HiChIP and two biological replicates for ChIP-Rx.<br>For our newly developed HiS-NET-seq, we obtained at least two biological replicate measurements for each condition. This number meets or exceeds the standard used for other high-resolution Pol II profiling methods (Nojima et al., 2015; Core et al., 2014). However, for biological conditions with naturally high biological variability (here: very short treatment time of 40 minutes for BRD4 degradation) we produced more replicates (four biological replicates).<br>For the dual luciferase reporter assay to examine enhancer activity of the DNA constructs each construct was measured at least in biological triplicates. For each biological replicate at least two technical replicates were measured with 7 Firefly and 5 Renilla readings per sample. This allows for statistical analysis and detection and removal of potential outliers and also meets the standards for this assay. For the proteomics data, five biological replicates were measured per condition, which meets or exceeds the number of biological replicates used for this assay and also allows for statistical analysis and detection and removal of potential outliers. |
| Data exclusions | No sample measurements were removed from the respective experiments. We removed signal regions from HiS-NET-seq, NET-seq, ChIP-Rx, and Hi-ChIP data as described in the corresponding method sections or figure legends. Signal masking from blacklisted regions is part of the standard data processing pipeline. |
| Replication | We included a suitable number of replicate measurements to ensure reproducibility. Only reproducible findings were derived from the data. HiChIP was performed in three biological replicates. ChIP-Rx was performed in two biological replicates. HiS-NET-seq was performed at least in two biological replicates. The dual luciferase reporter assay was performed at least in biological triplicate and technical duplicates. IP-MS experiments were performed with five biological replicates. |
| Randomization | Randomization was not required as all experiments and findings were derived from the same human K562 cell line. |
| Blinding | Blinding was not required as all experiments and findings were derived from the same human K562 cell line. |

# Behavioural & social sciences study design

All studies must disclose on these points even when the disclosure is negative.

| | |
|---|---|
| Study description | *Briefly describe the study type including whether data are quantitative, qualitative, or mixed-methods (e.g. qualitative cross-sectional, quantitative experimental, mixed-methods case study).* |
| Research sample | *State the research sample (e.g. Harvard university undergraduates, villagers in rural India) and provide relevant demographic information (e.g. age, sex) and indicate whether the sample is representative. Provide a rationale for the study sample chosen. For studies involving existing datasets, please describe the dataset and source.* |
| Sampling strategy | *Describe the sampling procedure (e.g. random, snowball, stratified, convenience). Describe the statistical methods that were used to predetermine sample size OR if no sample-size calculation was performed, describe how sample sizes were chosen and provide a rationale for why these sample sizes are sufficient. For qualitative data, please indicate whether data saturation was considered, and what criteria were used to decide that no further sampling was needed.* |
| Data collection | *Provide details about the data collection procedure, including the instruments or devices used to record the data (e.g. pen and paper, computer, eye tracker, video or audio equipment) whether anyone was present besides the participant(s) and the researcher, and whether the researcher was blind to experimental condition and/or the study hypothesis during data collection.* |
| Timing | *Indicate the start and stop dates of data collection. If there is a gap between collection periods, state the dates for each sample cohort.* |
| Data exclusions | *If no data were excluded from the analyses, state so OR if data were excluded, provide the exact number of exclusions and the rationale behind them, indicating whether exclusion criteria were pre-established.* |

| | |
|---|---|
| Non-participation | *State how many participants dropped out/declined participation and the reason(s) given OR provide response rate OR state that no participants dropped out/declined participation.* |
| Randomization | *If participants were not allocated into experimental groups, state so OR describe how participants were allocated to groups, and if allocation was not random, describe how covariates were controlled.* |

# Ecological, evolutionary & environmental sciences study design

All studies must disclose on these points even when the disclosure is negative.

| | |
|---|---|
| Study description | *Briefly describe the study. For quantitative data include treatment factors and interactions, design structure (e.g. factorial, nested, hierarchical), nature and number of experimental units and replicates.* |
| Research sample | *Describe the research sample (e.g. a group of tagged Passer domesticus, all Stenocereus thurberi within Organ Pipe Cactus National Monument), and provide a rationale for the sample choice. When relevant, describe the organism taxa, source, sex, age range and any manipulations. State what population the sample is meant to represent when applicable. For studies involving existing datasets, describe the data and its source.* |
| Sampling strategy | *Note the sampling procedure. Describe the statistical methods that were used to predetermine sample size OR if no sample-size calculation was performed, describe how sample sizes were chosen and provide a rationale for why these sample sizes are sufficient.* |
| Data collection | *Describe the data collection procedure, including who recorded the data and how.* |
| Timing and spatial scale | *Indicate the start and stop dates of data collection, noting the frequency and periodicity of sampling and providing a rationale for these choices. If there is a gap between collection periods, state the dates for each sample cohort. Specify the spatial scale from which the data are taken* |
| Data exclusions | *If no data were excluded from the analyses, state so OR if data were excluded, describe the exclusions and the rationale behind them, indicating whether exclusion criteria were pre-established.* |
| Reproducibility | *Describe the measures taken to verify the reproducibility of experimental findings. For each experiment, note whether any attempts to repeat the experiment failed OR state that all attempts to repeat the experiment were successful.* |
| Randomization | *Describe how samples/organisms/participants were allocated into groups. If allocation was not random, describe how covariates were controlled. If this is not relevant to your study, explain why.* |
| Blinding | *Describe the extent of blinding used during data acquisition and analysis. If blinding was not possible, describe why OR explain why blinding was not relevant to your study.* |

Did the study involve field work? ☐ Yes ☐ No

## Field work, collection and transport

| | |
|---|---|
| Field conditions | *Describe the study conditions for field work, providing relevant parameters (e.g. temperature, rainfall).* |
| Location | *State the location of the sampling or experiment, providing relevant parameters (e.g. latitude and longitude, elevation, water depth).* |
| Access & import/export | *Describe the efforts you have made to access habitats and to collect and import/export your samples in a responsible manner and in compliance with local, national and international laws, noting any permits that were obtained (give the name of the issuing authority, the date of issue, and any identifying information).* |
| Disturbance | *Describe any disturbance caused by the study and how it was minimized.* |

# Reporting for specific materials, systems and methods

We require information from authors about some types of materials, experimental systems and methods used in many studies. Here, indicate whether each material, system or method listed is relevant to your study. If you are not sure if a list item applies to your research, read the appropriate section before selecting a response.

## Materials & experimental systems

| n/a | Involved in the study |
|---|---|
| ☐ | ☒ Antibodies |
| ☐ | ☒ Eukaryotic cell lines |
| ☒ | ☐ Palaeontology and archaeology |
| ☒ | ☐ Animals and other organisms |
| ☒ | ☐ Clinical data |
| ☒ | ☐ Dual use research of concern |

## Methods

| n/a | Involved in the study |
|---|---|
| ☐ | ☒ ChIP-seq |
| ☒ | ☐ Flow cytometry |
| ☒ | ☐ MRI-based neuroimaging |

# Antibodies

Antibodies used

primary: BRD4 (Bethyl, catalog no. A301-985A50; 8 µg per ChIP, 10 µg per IP), GAPDH (Ambion, catalog no. 326548, 1:15,000 for WB), Histone H2B (A-6) (Santa Cruz Biotechnology, catalog no. sc-515808; 1:1,000 for WB), Histone H3K27ac (Abcam, catalog no. ab4729; 5 µg per HiChIP and 1:1000 for WB), HA tag (Cell Signaling Technology, catalog no. C29F4; 1:1,000 for WB), RPB1 Ser2-P (3E10) (Active Motif, catalog no. 61083; 1:1,000 for WB); secondary: IRDye® 800CW Goat anti-Rabbit IgG Secondary Antibody (LiCOR, catalog no. 926-32211; 1:15,000 for WB), IRDye® 800CW Goat anti-Mouse IgG Secondary Antibody (LiCOR, catalog no. 926-32210, 1:15000 for WB), IRDye® 800CW Goat anti-Rat IgG Secondary Antibody (LiCOR, catalog no. 926-32219, 1:15000 for WB), IRDye® 800CW Streptavidin antibody (LiCOR, catalog no. 925-32230, 1:15000 for WB).

Validation

The anti-BRD4 polyclonal antibody was raised in rabbit against an epitope consisting of the amino acids 1312 to 1362 of BRD4, that is, the C-terminal portion that is specific to BRD4-L but absent from other BET species. It is recommended by the manufacturer for WB, IP and IHC in human and mouse. Validation by the manufacturer included the parallel application of independent antibodies against the same target and the use in IHC and IP as complementary applications. The antibody was cited in 135 publications (according to the manufacturer).

The anti-GAPDH monoclonal antibody was raised in mouse against the purified full-length rabbit GAPDH molecule and is recommended for WB, IP, IHC, and ICC/IF in human, mouse, rabbit, rat, as well as non-mammalian vertebrates. For validation, the manufacturer employed the antibody for WB and IF as complementary applications and used a k.d. strategy to deplete the target, followed by IF. The antibody was cited by 500 publications (according to the manufacturer).

The anti-H2B monoclonal antibody was raised in mouse against the purified full-length human H2B and is recommended for WB, IP, IHC, and IF in human, mouse and rat. Validation by the manufacturer included use in IHC as a complementary application and WB upon overexpression of the target. The antibody was cited by 126 publications (according to CiteAb).

The anti-H3K27ac was raised in rabbit against a synthetic eptitope consisting of amino acids 1 to 100 of the human protein and is recommended for ChIP, WB, IF, and IHC in human, mouse, rat and cow. Validation by the manufacturer included the comparison with independent antibodies of the same target, ChIP-qPCR assays, IHC, peptide assays using peptides carrying different modifications, competition assays with the immunizing peptide, and assays using inhibitors reducing the histone modification. The antibody was cited by 1539 publications (according to the manufacturer).

The anti-HA tag polyclonal antibody was raised in rabbit against a synthetic peptide containing the HA epitope and is recommended for ChIP, IP, WB, IF, IHC, and flow cytometry. For validation, the manufacturer performed WB, IP, IHC, and flow cytometry in cell lines with or without expression of the tag, and did ChIP-qPCR upon treatment altering binding of the tagged protein. The antibody was cited by 1583 publications (according to the manufacturer).

The anti-RPB1 Ser2-P monoclonal antibody was raised in mouse against a peptide containing the serine 2-phosphorylated CTD sequence and is recommended for WB, ChIP, and IF in human and mouse. Validation by the manufacturer included the comparison of the ChIP-seq profile with that of other RPB1 phospho-forms, and IF. The antibody was cited by 40 publications (according to CiteAb).

The anti-Tubulin polyclonal antibody was raised in rabbit and a synthetic peptide conjugated to KLH derived from within residues 400 to the C-terminus of Human alpha Tubulin was used to generate this antibody. The antibody works for WB for HeLa, HEK-293, HepG2, Caco-2, NIH/3T3 and PC-12 whole cell lysates. For ICC/IF HeLa, Caco-2, NIH/3T3 and SV40LT-SMC cells were tested.The antibody was referenced in 331 publications (according to the manufacturers website)

Moreover, antibodies against BRD4 and the HA tag were validated by target degradation and immunoblotting or ChIP-Rx (see also Arnold et al., 2021).

# Eukaryotic cell lines

Policy information about cell lines and Sex and Gender in Research

Cell line source(s)

Human female K562 cells (catalog no. CCL-243) and murine male NIH/3T3 (catalog no. CRL-1658) cells were obtained from ATCC.

Authentication

All cell lines used were directly obtained from ATCC, a trusted vendor, in 2017. Only low-passage cells were used in experiments. Cell lines were not authenticated.

| | |
|---|---|
| Mycoplasma contamination | All cell lines were tested regularly, i. e., every 2 to 3 months, for mycoplasma contamination using a biochemical commercial assay (MycoAlert, Lonza, catalog no. LT07-318). Additionally, cells were preventively treated with Plasmocin (InvivoGen, catalog code ant-mpp). None of the cell lines tested mycoplasma-positive at any point. |
| Commonly misidentified lines (See ICLAC register) | None of the cell lines used in this study are considered commonly misidentified. |

## Palaeontology and Archaeology

| | |
|---|---|
| Specimen provenance | *Provide provenance information for specimens and describe permits that were obtained for the work (including the name of the issuing authority, the date of issue, and any identifying information). Permits should encompass collection and, where applicable, export.* |
| Specimen deposition | *Indicate where the specimens have been deposited to permit free access by other researchers.* |
| Dating methods | *If new dates are provided, describe how they were obtained (e.g. collection, storage, sample pretreatment and measurement), where they were obtained (i.e. lab name), the calibration program and the protocol for quality assurance OR state that no new dates are provided.* |

☐ Tick this box to confirm that the raw and calibrated dates are available in the paper or in Supplementary Information.

| | |
|---|---|
| Ethics oversight | *Identify the organization(s) that approved or provided guidance on the study protocol, OR state that no ethical approval or guidance was required and explain why not.* |

Note that full information on the approval of the study protocol must also be provided in the manuscript.

## Animals and other research organisms

Policy information about studies involving animals; ARRIVE guidelines recommended for reporting animal research, and Sex and Gender in Research

| | |
|---|---|
| Laboratory animals | *For laboratory animals, report species, strain and age OR state that the study did not involve laboratory animals.* |
| Wild animals | *Provide details on animals observed in or captured in the field; report species and age where possible. Describe how animals were caught and transported and what happened to captive animals after the study (if killed, explain why and describe method; if released, say where and when) OR state that the study did not involve wild animals.* |
| Reporting on sex | *Indicate if findings apply to only one sex; describe whether sex was considered in study design, methods used for assigning sex. Provide data disaggregated for sex where this information has been collected in the source data as appropriate; provide overall numbers in this Reporting Summary. Please state if this information has not been collected. Report sex-based analyses where performed, justify reasons for lack of sex-based analysis.* |
| Field-collected samples | *For laboratory work with field-collected samples, describe all relevant parameters such as housing, maintenance, temperature, photoperiod and end-of-experiment protocol OR state that the study did not involve samples collected from the field.* |
| Ethics oversight | *Identify the organization(s) that approved or provided guidance on the study protocol, OR state that no ethical approval or guidance was required and explain why not.* |

Note that full information on the approval of the study protocol must also be provided in the manuscript.

## Clinical data

Policy information about clinical studies

All manuscripts should comply with the ICMJE guidelines for publication of clinical research and a completed CONSORT checklist must be included with all submissions.

| | |
|---|---|
| Clinical trial registration | *Provide the trial registration number from ClinicalTrials.gov or an equivalent agency.* |
| Study protocol | *Note where the full trial protocol can be accessed OR if not available, explain why.* |
| Data collection | *Describe the settings and locales of data collection, noting the time periods of recruitment and data collection.* |
| Outcomes | *Describe how you pre-defined primary and secondary outcome measures and how you assessed these measures.* |

## Dual use research of concern

Policy information about dual use research of concern

### Hazards

Could the accidental, deliberate or reckless misuse of agents or technologies generated in the work, or the application of information presented in the manuscript, pose a threat to:

No | Yes
- [ ] [ ] Public health
- [ ] [ ] National security
- [ ] [ ] Crops and/or livestock
- [ ] [ ] Ecosystems
- [ ] [ ] Any other significant area

## Experiments of concern

Does the work involve any of these experiments of concern:

No | Yes
- [ ] [ ] Demonstrate how to render a vaccine ineffective
- [ ] [ ] Confer resistance to therapeutically useful antibiotics or antiviral agents
- [ ] [ ] Enhance the virulence of a pathogen or render a nonpathogen virulent
- [ ] [ ] Increase transmissibility of a pathogen
- [ ] [ ] Alter the host range of a pathogen
- [ ] [ ] Enable evasion of diagnostic/detection modalities
- [ ] [ ] Enable the weaponization of a biological agent or toxin
- [ ] [ ] Any other potentially harmful combination of experiments and agents

# ChIP-seq

## Data deposition

- [x] Confirm that both raw and final processed data have been deposited in a public database such as GEO.
- [ ] Confirm that you have deposited or provided access to graph files (e.g. BED files) for the called peaks.

| | |
|---|---|
| Data access links<br>*May remain private before publication.* | https://www.ncbi.nlm.nih.gov/geo/query/acc.cgi?acc=GSE214594 |
| Files in database submission | GRCh38_p12.MRA109.FE.MRA112.bw<br>GRCh38_p12.MRA115.FE.MRA116.bw<br>GRCh38_p12.MRA114.FE.MRA116.bw<br>GRCh38_p12.MRA111.FE.MRA112.bw<br>GRCm38_p6.MRA115.FE.MRA116.bw<br>GRCm38_p6.MRA109.FE.MRA112.bw<br>GRCh38_p12.MRA110.FE.MRA112.bw<br>GRCm38_p6.MRA114.FE.MRA116.bw<br>GRCh38_p12.MRA113.FE.MRA116.bw<br>GRCm38_p6.MRA111.FE.MRA112.bw<br>GRCm38_p6.MRA113.FE.MRA116.bw<br>GRCm38_p6.MRA110.FE.MRA112.bw<br>GRCh38_p12.MRA116.rpm.bw<br>GRCm38_p6.MRA116.rpm.bw<br>GRCh38_p12.MRA112.rpm.bw<br>GRCm38_p6.MRA112.rpm.bw<br>MRA116_R2.fastq.gz<br>MRA116_R1.fastq.gz<br>MRA115_R2.fastq.gz<br>MRA115_R1.fastq.gz<br>MRA114_R2.fastq.gz<br>MRA114_R1.fastq.gz<br>MRA113_R2.fastq.gz<br>MRA113_R1.fastq.gz<br>MRA112_R2.fastq.gz<br>MRA112_R1.fastq.gz<br>MRA111_R2.fastq.gz<br>MRA111_R1.fastq.gz<br>MRA110_R2.fastq.gz<br>MRA110_R1.fastq.gz<br>MRA109_R2.fastq.gz<br>MRA109_R1.fastq.gz |

Genome browser session (e.g. UCSC) | IGV v2.8 session available at https://oc-molgen.gnz.mpg.de/owncloud/s/F53yoFzbbxJty8K.

## Methodology

Replicates: ChIP-Rx samples were measured in biological duplicates for each condition. Pearson's correlation clustering are shown in Supplementary Figure 19.

Sequencing depth: All ChIP-Rx samples were sequenced in paired-end mode with a respective sequencing length of 100 bp. We sequenced between 60-97 million fragments for each sample.

Antibodies: See "Antibodies" section above.

Peak calling parameters: We used Bowtie2 v2.3.5.1 to align the sequencing reads to a joint reference which consisted of the human (GRCh38.p12) and mouse (GRCm38.p6) reference genomes using the paired-end mode with the parameter -k 1. As recommended by the MACS2 developers, potential PCR duplicates were marked using PICARDs v2.24.2 markDuplicates function.
MACS2 identified peaks using the following parameters: -B --SPMR -f BAMPE -g 4.57e+09 --nomodel --extsize 150 --call-summits.

Data quality: Consensus peaks were identified by the DiffBind v3.0.15 package as described in the respective methods section of the manuscript.

Software: Bowtie2 v2.3.5.1, PICARD v2.24.2, MACS2 v2.2.7.1, deepTools2 v3.2.1

# Flow Cytometry

## Plots

Confirm that:

- [ ] The axis labels state the marker and fluorochrome used (e.g. CD4-FITC).
- [ ] The axis scales are clearly visible. Include numbers along axes only for bottom left plot of group (a 'group' is an analysis of identical markers).
- [ ] All plots are contour plots with outliers or pseudocolor plots.
- [ ] A numerical value for number of cells or percentage (with statistics) is provided.

## Methodology

Sample preparation: *Describe the sample preparation, detailing the biological source of the cells and any tissue processing steps used.*

Instrument: *Identify the instrument used for data collection, specifying make and model number.*

Software: *Describe the software used to collect and analyze the flow cytometry data. For custom code that has been deposited into a community repository, provide accession details.*

Cell population abundance: *Describe the abundance of the relevant cell populations within post-sort fractions, providing details on the purity of the samples and how it was determined.*

Gating strategy: *Describe the gating strategy used for all relevant experiments, specifying the preliminary FSC/SSC gates of the starting cell population, indicating where boundaries between "positive" and "negative" staining cell populations are defined.*

- [ ] Tick this box to confirm that a figure exemplifying the gating strategy is provided in the Supplementary Information.

# Magnetic resonance imaging

## Experimental design

Design type: *Indicate task or resting state; event-related or block design.*

Design specifications: *Specify the number of blocks, trials or experimental units per session and/or subject, and specify the length of each trial or block (if trials are blocked) and interval between trials.*

Behavioral performance measures: *State number and/or type of variables recorded (e.g. correct button press, response time) and what statistics were used to establish that the subjects were performing the task as expected (e.g. mean, range, and/or standard deviation across subjects).*

## Acquisition

Imaging type(s) | *Specify: functional, structural, diffusion, perfusion.*

Field strength | *Specify in Tesla*

Sequence & imaging parameters | *Specify the pulse sequence type (gradient echo, spin echo, etc.), imaging type (EPI, spiral, etc.), field of view, matrix size, slice thickness, orientation and TE/TR/flip angle.*

Area of acquisition | *State whether a whole brain scan was used OR define the area of acquisition, describing how the region was determined.*

Diffusion MRI ☐ Used ☐ Not used

## Preprocessing

Preprocessing software | *Provide detail on software version and revision number and on specific parameters (model/functions, brain extraction, segmentation, smoothing kernel size, etc.).*

Normalization | *If data were normalized/standardized, describe the approach(es): specify linear or non-linear and define image types used for transformation OR indicate that data were not normalized and explain rationale for lack of normalization.*

Normalization template | *Describe the template used for normalization/transformation, specifying subject space or group standardized space (e.g. original Talairach, MNI305, ICBM152) OR indicate that the data were not normalized.*

Noise and artifact removal | *Describe your procedure(s) for artifact and structured noise removal, specifying motion parameters, tissue signals and physiological signals (heart rate, respiration).*

Volume censoring | *Define your software and/or method and criteria for volume censoring, and state the extent of such censoring.*

## Statistical modeling & inference

Model type and settings | *Specify type (mass univariate, multivariate, RSA, predictive, etc.) and describe essential details of the model at the first and second levels (e.g. fixed, random or mixed effects; drift or auto-correlation).*

Effect(s) tested | *Define precise effect in terms of the task or stimulus conditions instead of psychological concepts and indicate whether ANOVA or factorial designs were used.*

Specify type of analysis: ☐ Whole brain ☐ ROI-based ☐ Both

Statistic type for inference
(See Eklund et al. 2016) | *Specify voxel-wise or cluster-wise and report all relevant parameters for cluster-wise methods.*

Correction | *Describe the type of correction and how it is obtained for multiple comparisons (e.g. FWE, FDR, permutation or Monte Carlo).*

## Models & analysis

| n/a | Involved in the study |
|---|---|
| ☐ | ☐ Functional and/or effective connectivity |
| ☐ | ☐ Graph analysis |
| ☐ | ☐ Multivariate modeling or predictive analysis |

Functional and/or effective connectivity | *Report the measures of dependence used and the model details (e.g. Pearson correlation, partial correlation, mutual information).*

Graph analysis | *Report the dependent variable and connectivity measure, specifying weighted graph or binarized graph, subject- or group-level, and the global and/or node summaries used (e.g. clustering coefficient, efficiency, etc.).*

Multivariate modeling and predictive analysis | *Specify independent variables, features extraction and dimension reduction, model, training and evaluation metrics.*
